# A novel simulation-based approach to training for recruitment of older adults to clinical trials

**DOI:** 10.1186/s12874-022-01643-4

**Published:** 2022-06-28

**Authors:** Harriet Fisher, Sondra Zabar, Joshua Chodosh, Aisha Langford, Chau Trinh-Shevrin, Scott Sherman, Lisa Altshuler

**Affiliations:** 1 Division of General Internal Medicine and Clinical Innovation, NYU Grossman School of Medicine (NYUGSOM), New York, USA; 2 Department of Population Health, NYU Grossman School of Medicine (NYUGSOM), New York, USA; 3 Division of Geriatric Medicine, NYU Grossman School of Medicine (NYUGSOM), New York, USA

**Keywords:** Simulation, Participation, Clinical Trials, Older Adults, Informed Consent, OSCE, GOSCE, Recruitment

## Abstract

**Background:**

The need to engage adults, age 65 and older, in clinical trials of conditions typical in older populations, (e.g. hypertension, diabetes mellitus, Alzheimer’s disease and related dementia) is exponentially increasing. Older adults have been markedly underrepresented in clinical trials, often exacerbated by exclusionary study criteria as well as functional dependencies that preclude participation. Such dependencies may further exacerbate communication challenges. Consequently, the evidence of what works in subject recruitment is less generalizable to older populations, even more so for those from racial and ethnic minority and low-income communities.

**Methods:**

To support capacity of research staff, we developed a virtual, three station simulation (Group Objective Structured Clinical Experience—GOSCE) to teach research staff communication skills. This 2-h course included a discussion of challenges in recruiting older adults; skills practice with Standardized Participants (SPs) and faculty observer who provided immediate feedback; and debrief to highlight best practices. Each learner had opportunities for active learning and observational learning. Learners completed a retrospective pre-post survey about the experience. SP completed an 11-item communication checklist evaluating the learner on a series of established behaviorally anchored communication skills (29).

**Results:**

In the research staff survey, 92% reported the overall activity taught them something new; 98% reported it provided valuable feedback; 100% said they would like to participate again. In the SP evaluation there was significant variation: the percent well-done of items by case ranged from 25–85%.

**Conclusions:**

Results from this pilot suggest that GOSCEs are a (1) acceptable; (2) low cost; and (3) differentiating mechanism for training and assessing research staff in communication skills and structural competency necessary for participant research recruitment.

**Supplementary Information:**

The online version contains supplementary material available at 10.1186/s12874-022-01643-4.

## Background

Despite an increasing need to recruit older clinical trial participants, adults over 65, in particular, are markedly underrepresented. Research participation has steadily declined over the past 30 years [1–3]. Racial or ethnic minorities are particularly underrepresented in clinical research [4–6]. Consequently, the evidence of what treatment and intervention strategies work in participant recruitment is less generalizable to older populations, and even less so for those from racial and ethnic minority and low-income communities. This is particularly troubling for clinical trials that study conditions such as hypertension, diabetes mellitus, Alzheimer’s disease and related dementia typical in older populations and more prevalent among minority groups [7, 8].

Establishing trust and rapport are fundamental to effective recruitment of older adults into research [9]; Atwood found staff rapport was one of the top four motivations for participation among older adults [10]. Dimensions of research staff, such as communication, flexibility, respect, and appreciation are reported to be among the leading reasons why older adults ages 65 and older remained in studies [11, 12]. Yet structural competency – i.e. the capacity of health professionals to understand the psychosocial and health context of older adults – is limited among research teams [12]. One barrier to recruitment is inattention to specific characteristics that older age participants face and that differ from younger age populations. Such characteristics necessitate different recruitment strategies [4, 13]. These include allowing verbal responses for those with manual dexterity challenges, allotting extra time for those with slower cognitive processing speed, and employing written materials for those with hearing impairment, as some salient examples [4]. Research staff are often tasked with recruitment and need communication skills to respond to these challenges. Nishimura et al. suggest that training to improve communication skills might be important in enhancing participant consent [14].

While studies have suggested that “education,” defined as materials or classroom learning, does not increase the efficacy of recruiters or research assistants in recruiting individuals to trials, there is little information on the feasibility or potential efficacy of simulation-based educational experiences in training these recruiters [15]. In a 2013, meta-analysis of 45 interventions to improve recruitment to clinical trials, just two considered interventions aimed at the recruiters themselves [14]. Of those two, one trial increased recruitment, one trial had no impact, but both educational interventions focused only on didactic learning. Meta-analysis authors concluded that there was a crucial knowledge gap in recruitment interventions aimed at recruiters [15]. Simulation and performance based training with trained actors playing standardized patients are used in medical education to teach communication skills. The Group Objective Structured Clinical Experience (GOSCE) is a simulation-based experience in which learners move in groups to practice and then reflect on their communication skills. GOSCEs address that critical gap in recruitment interventions aimed at recruiters by aiming an intervention at recruiters. In an effort to address low recruitment of older adults, we sought to assess the acceptability of implementing a simulated educational experience, one of the most widely used methods of assessing and developing aspects of clinical competency in healthcare education, for research assistants [16].

We developed a quality improvement project that consisited of a three station GOSCE where research staff practiced a recruitment protocol for a hypothetical clinical trial (in which older adults would receive weekly visits with a puppy) using simulated older adult participants. Table 1 outlines the GOSCE structure. Through the GOSCE we sought to enhance recruiters’ communication skills and capacity to engage, build trust and ultimately enroll older participants into research. We chose this simulation methodology based on evidence that practicing these skills and receiving immediate feedback is a high yield strategy for strengthening communication skills in physician and non-physician healthcare workers [17–19]. Our objective was to assess the acceptability of GOSCEs to train research staff in best practices for recruiting and consenting older adults to clinical trials.

**Table 1:** Overview of GOSCE structure

Timing	GOSCE Activity	GOSCE Participants
*30 min*	Orientation to GOSCE: participants and faculty introduce themselves; discussion on challenges and facilitators to obtaining consent and a model for effective recruitment	Two medical education faculty members; learners 1–9
*10 min*	*Case 1*: Group 1 (Participants 1, 2, 3, faculty member 1, SP) *Case 2*: Group 2 (Participants 4, 5, 6, faculty member 2, SP) *Case 3*: Group 3 (Participants 7, 8, 9, faculty member 3, SP)	Participants 1,4,7 complete encounter w/ SP; other participants and faculty member observe
*10 min*	Faculty member leads 10 min debrief w/ respective group; SP completes 11-item communication skills checklist	
*10 min*	*Case 2*: Group 1 (Participants 1, 2, 3, faculty member 2) *Case 3*: Group 2 (Participants 4, 5, 6, faculty member 3) *Case 1*: Group 3 (Participants 7, 8, 9, faculty member 1)	Participants 2, 5, 8 complete encounter w/ SP; other participants and faculty member observe
*10 min*	Faculty member leads 10 min debrief w/ respective group; SP completes 11-item communication skills checklist	
*10 min*	*Case 3*: Group 1 (Participants 1, 2, 3, faculty member 3) *Case 1*: Group 2 (Participants 4, 5, 6, faculty member 1) *Case 2*: Group 3 (Participants 7, 8, 9, faculty member 2)	Participants 3, 6, 9 complete encounter w/ SP; other participants and faculty member observe
*10 min*	Faculty member leads 10 min debrief w/ respective group; SP completes 11-item communication skills checklist	
*10 min*	Faculty lead full group debrief highlight best practices and help integrate new approaches into behavioral repertoires; share handout on recruitment best practices	Two medical education faculty members; learners 1–9
(post-GOSCE)	Immediately following GOSCE participants receive link to an anonymous, 36-item survey	Retrospective Pre/Post Survey

## Materials and methods

GOSCEs are a more cost-and resource-effective [20, 21] variation on the traditional objective structured clinical experience (OSCE), which is a standard component in undergraduate and graduate medical education curricula and is used in many healthcare fields, including midwifery [22], physical and occupational therapy [23], nursing [24], and research [22, 25]. In a GOSCE learners move in *groups* through stations. In each station, one group member has the opportunity to lead the interaction with the standardized patient (a professional actor trained to play a patient), while others have a role observing [26, 27]. After the encounter, there is an opportunity for standardized patient (SP) perspective, peer debrief and feedback from a faculty observer which strengthens the GOSCE’s value as a formative assessment tool [20, 21, 28, 29]. This model, of concrete experience (with SP) followed by reflective observation (with the group and faculty member) is modeled after David Kolb’s experiential learning theory [30].

Two experienced medical educators with expertise in performance-based training and assessment designed the quality improvement, GOSCE experience with input from an IRB expert, a geriatrician, and a PhD researcher specializing in recruitment and retention in clinical trials, in consultation with leaders of community organizations for older adults. The scenarios for the simulated recruitment of older adults were 1) a Black woman with hearing impairment 2) a white woman and her family member both present with differing views on research participation, and 3) a Black man with concerns about participating given a history of racism in medical research. Cases were developed based on a literature review of facilitators and barriers to clinical trial recruitment and a focus group of 5 research staff who regularly recruit older adults to clinical trials. 4 SPs (one for each case, with two SPs for one case) were recruited from the pool of SPs employed by the medical school; they received 4 h of training (2 on the case portrayal, 2 on the checklist completion) by a physician with experience in performance-based assessment. Each item on the checklist is scored on a 3-point scale (not done, partly done and well done, each with a behavioral anchor describing the point). For example, “did not discuss risks,” “discussed risks BUT did not check for understanding or questions,” discussed risks AND checked for understanding or questions.’ The checklist is the standard model used in medical education at our site over the past 15 years and has good reliability and validity [31]. Competencies we aimed to assess include: building trust and rapport with participant; assessing understanding and capacity to consent; and presenting information. Cases were shared with experts in the clinical trial recruitment field and modified accordingly until expert consensus on the cases was reached.

In this GOSCE, the convenience sample included 45 research staff at a large, urban hospital, both staff who routinely recruit older adults to participate in clinical trials and staff who are involved in recruitment training and strategy. The GOSCE was conducted five times, each with 9 participants. The total cost of each GOSCE was 800 US dollars. GOSCE participants had been recruiters for less than two years. Using a virtual conferencing platform, this 2-h GOSCE included three sections: 1) a 30 min orientation to GOSCE with discussion on challenges and facilitators to obtaining consent and a model for effective recruitment; 2) three 20 min GOSCE stations with Standardized Participants (SPs) with 10 min for the interview and 10 min for immediate feedback; and 3) a 25 min group debrief to review experiences, highlight best practices and help integrate new approaches into behavioral repertoires. After the initial orientation, Research staff rotated in groups of three through three stations, each with an SP whom they needed to recruit to the “trial. An observing faculty member provided immediate feedback after this simulated recruitment effort and led a debrief with all three members of the group. During this debrief, the SP completed an 11-item communication checklist evaluating the learner on a series of behaviorally anchored communication skills; after finishing the checklist, the SP would join the debrief and share additional feedback based on the checklist. Each learner had opportunities for active learning and observational learning. After these encounters, the group came back together with all the faculty observers for a group discussion and debrief. A handout on best practices, developed by the team of medical educators and researcher with experience in clinical recruitment, was shared for participants to use in their future work.

The program was evaluated by research staff through a 36-question survey including retrospective pre-post items (i.e. learners completed the survey after the GOSCE reflecting on skills before and after GOSCE) that used 3 and 4-point Likert scale questions (i.e. not at all skilled to very skilled, low educational value to high educational value). Free text questions about experience with the cases and lessons learned from the training were also included. Questions addressed: 1) self-assessed change in skill after the workshop; 2) new discoveries in recruitment; and 3) overall educational value. The survey was adapted from a standard educational evaluation survey used in simulation training at our institution to elicit acceptability, relevance and change in knowledge and attitudes. Effectiveness of program was evaluated through Kirkpatrick’s training evaluation model Level 1 (Reaction) and Level 2 (Learning) [32].

This survey was distributed through an anonymous link post-GOSCE. None of the faculty members involved in the cases and feedback session are the supervisors of any of the participants of the GOSCE. Descriptive statistics and a nonparametric sign test for median differences on changes in self-reported skills were computed for survey items. Free text responses were assessed using a qualitative thematic analysis.

## Results

The curriculum was delivered as planned, but given the advent of the COVID-19 pandemic, the GOSCE was switched to a remote (zoom) platform in order to be able to conduct safely. It was conducted three times: July 2020, October 2020, and April 2021. Ninety-one percent of research staff (41/45) completed the 36-item survey. Research staff recruit at a large, urban hospital with recruitment experience between 0 months and 2 years and represent a broad disciplines and range of research projects. Of these, 93% (38/41 reported the overall activity taught them something new; 98% (40/41) reported it provided valuable feedback; 100% (41/41) said they would like to participate again. 56% (23/41) percent reported perceived improvement in their recruitment skills as a result of the workshop.

As outlined in Table 2, research staff reported that these cases were similar to real life (range: 56–73% had encountered similar situation to the case they participated in) and they found all cases had high or moderate educational value in strengthening their skills. Representative quotes from the survey regarding the GOSCE’s educational value are also included in Table 2; these were identified through a brief thematic analysis conducted by two medical education researchers. The case learners were least familiar with was also the case they rated as having the highest educational value. Indeed, just 56% of research staff had experiences of the third case, in which the SP is hesitant about participating in research given the history of racism in medicine and his brother’s experience of medical neglect, but 82% of research staff reported it was of high educational value.

**Table 2:** Evaluation by research staff of group objective structured clinical experience

Case Information	Case 1: Hearing impairment			Case 2: patient and family member w/ conflicting views			Concern given history of racism in medicine		
Encountered similar situations (Yes / No)	Had encountered similar situations = 30/41 (73%)			Had encountered similar = 24/41 (58%)			Had encountered similar = 23/41 (56%)		
Educational Value of Station	Low	Moderate	High	Low	Moderate	High	Low	Moderate	High
	0	11	30	0	12	25	0	5	34
Most difficult part of case (open ended response)	“Establishing rapport with the patient and being cognizant of her communication issues without making it seem like I was talking down to her or making her feel bad about the issues we were having.”			“Making sure that the subject felt heard while remaining on track with explaining the risks and benefits”			“Remaining culturally sensitive, acknowledging that there are differences between the experiences of different groups (depending on SES, ethnicity, sexual orientation, etc.) and being as empathetic as possible to the pt's experiences “		

Seven research staff rated their pre-GOSCE skills as “very skilled” by 7 research staff; 23 rated themselves as “somewhat skilled”; 8 as “not very skilled”; and 3 as “not at all skilled”. Post-GOSCE, 20 rated themselves as “very skilled” and 21 rated skills as “somewhat skilled.” Self-rated skills improved for 23 of 41 research staff, while 18 research staff pre- and post-ratings were unchanged. An exact sign test was used to compare pre and post ratings. Scores post-GOSCE had a significantly different median increase compared to pre-GOSCE, *p* = 0.0001.

In the survey, participants listed their key learning points that aligned with the aims of the GOSCE. Table 3 outlines themes that emerged from these primary learning points. These were identified through an open-coded thematic analysis conducted by two medical education researchers. Themes included: learning about other, learning about self, specific behaviors to change, and learning by doing.

**Table 3:** Themes from Participants reports of “three take home points from this group objective structure clinical experience program”

Learning about other
1. How through voicing the feelings of the participant, they feel more heard 2) To have the patient "teach back" to me and summarize what I had told them. 3) How to utilize technology to better connect with patients
2. Challenges in recruitment of older adults 2) being culturally appropriate and self-aware when recruiting 3) and how to interact with caregivers
3. If a patient is having issues, it's better to address them even though it may be uncomfortable to do so because it will provide you with insight about how to help 2) it's important to converse casually with the patient in order to establish trust and a friendly demeanor while also making sure to keep the conversation relevant to the purpose 3) there's value in establishing an emotional connection with people who might have doubts or anxiety
4. The importance of being precise and confident when we speak to a patient 2) Authenticity and genuineness help build patient rapport 3) Trying to slow down when I speak because speaking too fast can create a gap in communication with patients, so it is important to make sure the patient is hearing you well
Learning about self
1. It is okay to slow down 2) Acknowledge the person's feelings 3) Have them summarize what was said
2. Listen carefully and tailor my talk to what the person is saying 2) empathize with someone and addressing someone's emotions, 3) and to just have a conversation with someone (there is no one concrete way of consenting, etc.)
3. Interview skills 2) Cultural sensitivity 3) Better communication skills
Specific behaviors to change
1. Repeat yourself loud and clear 2) Don’t get flustered 3) Keep calm and smile
2. acknowledge the "elephants in the room" + name the issues that are present 2) check for comprehension 3) stay mindful of wording/body language
3. Engage in active listening 2) Always be culturally sensitive 3) Be calm, genuine, and transparent
4. Come prepared with alternative means of communication to deal with zoom—e.g. visuals like a document that can be shared via zoom, items to hold up to the camera, using the chat feature to type messages, etc. 2) Come prepared to help troubleshoot technology to deal with zoom—e.g. helping someone set up headphones, control volume, etc. 3) Prepare sufficiently with background knowledge so that surprise questions / curveballs are more easily answered
5. Need to do a thorough prescreening before interview
6. I will always try to take time to build trust and personal connection so the patient feels seen and heard. 2) When participants may feel frustrated, it is ok and helpful to name that feeling in order to move beyond it. 3) Patience and understanding of the participant's situation is critical
7. Not only have empathy but show it through my body language/facial expressions 2) Confirm understanding instead of just asking "Do you understand?" 3) Acknowledge people’s feelings and overtly ask about them
8. patient 2) Slow speaking 3) Empathy
Benefit of learning from doing
1. Feedback is important 2) receiving advise from experienced physicians is great 3) mock cases are a great way to learn new strategies and improve your recruiting

Due to internet challenges at the first GOSCE limiting completion of checklists, 39 of 45 communication checklists of participant performance during the GOSCE station (9 participants at each of 5 sessions) were completed by the SPs. Research staff performance on the 11-item communication checklist is reported in Table 4. These behaviorally anchored communication checklist items (Table 4) were developed by this team of medical educators and have demonstrated reliability [31]. There was a range of scores: the % well-done of items by case ranged from 25–85%. For example, SPs reported that 85% of research staff “did not interrupt and allowed time to express thoughts fully” 25% checked your understanding through specific questioning and/or asking you to repeat back information.

**Table 4:** Research staff performance on behaviorally anchored checklist as assessed by standardized patients

Domain	Checklist Item (Mean % Well Done)	Racism Case (*N* = 15)	Family member (*N* = 12)	Hearing (*N* = 12)	All Cases (*N* = 39)	Domain Mean % Well Done (SD)	Well Done Behavioral Anchor
		(Mean % Well Done)					
Relationship Develop	Communicated concern or intention to help	60%	83%	67%	69%	67% (6.1)	Actions and words conveyed intention to help/concern
	Non-Verbal Behavior enriched communication (e.g. eye contact, posture)	60%	83%	25%	56%		Non-verbal behavior facilitated effective communication
	Acknowledged emotions/feelings appropriately	67%	75%	50%	64%		Acknowledged and responded to your emotions in ways that made you feel better
	Was accepting/non-judgmental	73%	92%	58%	74%		Made comments and expressions that demonstrated respect
	Used words you understood and/or explained jargon	60%	92%	58%	69%		Provided no opportunity for misunderstanding by avoiding or spontaneously explaining jargon
Patient Education	Asked questions to see what you understood	47%	33%	25%	42%	47% (7.8)	Checked your understanding through specific questioning and/or asking you to repeat back information
	Provided clear explanations/information	53%	67%	50%	56%		Provided small bits of information at a time and summarized to ensure understanding
	Collaborated with you to identify and decide on possible next steps/plan	53%	17%	25%	33%		Elicited your views on next steps, shared her/his ideas, and mutually developed plan of action
Patient Satisfaction	Answered or addressed all of my questions/concern	47%	92%	75%	69%	65% (4.2)	Answered/addressed all of your questions/concern
	Took a personal interest in you; treated you as a person	60%	75%	50%	62%		Took an active personal interest in you
Information Gathering	Allowed you to talk without interrupting	80%	83%	92%	85%	85%	Did not interrupt and allowed time to express thoughts fully

## Discussion

The implementation of this GOSCE provides a novel methodology for increasing effective recruitment of older adults – but broadly all people – to clinical trials by aiming to increase recruitment through training recruiters (11). Results from this pilot suggest that GOSCEs are a (1) acceptable; (2) low cost; and (3) differentiating mechanism for training research staff in communication skills and structural competency necessary for participant research recruitment and identifying those that may need additional practice. This educational methodology is familiar to medical education, but novel for training those who recruit participants for clinical trials and addresses an important gap around recruitment training in the literature [25] (29). This data suggests that GOSCEs are acceptable trainings for research assistants and can be implemented in other settings to provide dynamic communication skills training.

Based on the retrospective pre-post, GOSCEs are feasible for training research staff in simulated clinical scenarios with which research staff reported they had experience; indeed, learners found this to be an educationally valuable experience (100% reported it was of high or moderate educational value, Table 2) and one that reflected situations they experience in practice (56–73% had encountered similar situation). This retrospective survey is standard practice in medical education and is a useful alternative to a pre-test where learners often over or under-estimate their abilities in (i.e. they don’t know what they don’t know), but pre-post tests are limited in that it may be influenced by social desirability and recall bias [33] (30). We selected three case scenarios based on interviews, literature review and relevance for these research staff, but this methodology is highly adaptable to a wide range of sensory, cultural, and individual challenges. GOSCEs are highly adaptable to the specific recruitment needs of a clinical trial and this work suggests they are useful for educating research staff.

GOSCEs are less expensive and time-intensive than some may expect. Simulation-based education is quite feasible, especially given that the vast majority of academic medical centers have simulation centers with whom one can partner to conduct this work. In addition, if SPs are not available, role-play can serve as a substitute for SPs. The same behaviorally anchored checklist to inform feedback to the learner can be used. For reference, the overall cost of training and employing 4 SPs for this GOSCE program (4 h training/actor + 2 h of GOSCE case/actor × 5 iterations) for the 45 research staff was approximately $2200.00. For comparison, conducting individual OSCEs for 45 research staff would cost at least $3400.00. These data suggest this innovation is an acceptable, low cost strategy for training research staff.

SP assessment of research staff identified variation in communication skills and suggested areas for further reinforcement to improve recruitment skills. The range of percent-well-done in SP evaluations indicates that GOSCEs can distinguish between higher and lower performing learners and provide behaviorally specific areas for feedback. In particular, the range of % well-done of items by case ranged from 25–85%. The lowest percent well done (25%) scores were the case where the SP had hearing loss, suggesting higher need for remediation skills that around accounts for auditory impairment of potential research participants. This variation in skills is notable given communication skills play a significant role in recruitment: a 2013 study that surveyed researchers and community members on the same questions about their practices and preferences during the informed consent process found that the ability to engage in one-on-one discussion about the study was most important for researchers and second most important (after take home materials) for community members, suggesting that identifying and strengthening skills around communication is central to effectively training those who recruit and consent participants to clinical trials [34].

While we are limited by lacking data on the impact this GOSCE had on participants’ overall recruitment success, we believe this interactive approach is likely to be more fruitful than the traditional classroom learning – a strategy that has not been found to be effective in increasing recruiters’ ability to recruit [35]. We also limited in generalizability due to the sample and setting, potential limitations of the methods used to evaluate the GOSCE.

## Conclusions

This pilot suggests that GOSCEs are a (1) acceptable; (2) low cost; and (3) differentiating mechanism for training research staff in communication skills and structural competency necessary for participant research recruitment. In addition, when surveyed, participants overwhelmingly felt it taught them new information and provided valuable feedback. Given the relatively low cost of implementing this program, we believe it has potential for researchers and research institutions looking to increase the participation of older adults in their research and clinical trials. Our next steps are to gather data on the impact of GOSCE training on research staff behavior during recruitment and consenting, and on research staff success in recruitment and research participants.

## Supplementary Information

**Additional file 1.**

## Data Availability

The datasets used and/or analyzed during the current study are available from the corresponding author on reasonable request.
