# Supplementary material for: A novel simulation-based approach to training for recruitment of older adults to clinical trials
Source: BMC Med Res Methodol. 2022 Jun 28;22:180. doi: 10.1186/s12874-022-01643-4 (PMC9238219; doi:10.1186/s12874-022-01643-4)
Supplement: Supplementary file 1 — Additional file 1. [file 12874_2022_1643_MOESM1_ESM.docx]

Checklist items:

| Introduces self and purpose | 0, 0, Did not introduce self or purpose \| 1,1, Introduced self OR purpose \| 2,2, Introduced self AND purpose |
| --- | --- |
| Allowed you to talk without interrupting | 0, 0, Interrupted \| 1,1, Did not interrupt BUT cut responses short, not enough time \| 2,2, Did not interrupt; Allowed to express thoughts fully |
| Communicated concern or intention to help | 0, 0, Did not communicate intention to help/concern \| 1,1, Words OR actions conveyed intention to help/concern \| 2,2, Words AND actions conv |
| Non-verbal behavior enriched communication (e.g., eye contact, posture) | 0, 0, Non-verbal behavior was negative OR interfered with communication \| 1,1, Non-verbal behavior demonstrated attentiveness \| 2,2, Non-verbal behavior facilitated effective communication |
| Acknowledged emotions/feelings appropriately | 0, 0, DID NOT acknowledge emotions/feelings \| 1,1, Acknowledged emotions/feelings \| 2,2, Acknowledged and responded in ways that made you feel better |
| Was accepting/non-judgmental | 0, 0, Made judgmental comments OR facial expressions \| 1,1, Did not express judgment but did not demonstrate respect \| 2,2, Made comments and expressions that demonstrated respect |
| Used words you understood and/or explained jargon | 0, 0, Consistently used jargon WITHOUT further explanation \| 1,1, Sometimes used jargon AND did not explain it \| 2,2, Explained jargon when used OR avoided completely |
| Asked questions to see what you understood (checked your understanding) | 0, 0, Did not check for understanding \| 1,1, Asked if you had any questions BUT did not check for understanding \| 2,2, Assessed understanding by checking in throughout |
| Provided clear explanations/information | 0, 0, Gave confusing/no explanations- made it impossible to understand \| 1,1, Info was somewhat clear BUT still led to some difficulty in understanding \| 2,2, Provided small bits of info AND summarized to make sure clear |
| Collaborated with you in identifying possible next steps/plan | 0, 0, Told you next steps/plan (OR no next steps/plan) \| 1,1, Told you next steps THEN asked your views \| 2,2, Discussed options THEN mutually developed plan |
| Answered or addressed all your questions or concerns | 0, 0, Answered/addressed only a few of your most central questions \| 1,1, Answered/addressed many of your questions or concerns \| 2,2, Answered/addressed all of your questions or concerns |
| Took a personal interest in you; treated you as a person | 0, 0, Did not see you as a person \| 1,1, Viewed you as a person, but did not take personal interest \| 2,2, Took an active personal interest in you |

| Case 1: | |
| --- | --- |
| 1. After greeting, asks permission to continue | 0, 0, Did not ask/address permission to continue \| 1,1, Assumed/narrated permission to continue (e.g. ”I am going to continue explaining…”  \| 2,2, Asked permission to continue |
| 1. Uses individual’s name | 0, 0, Did not use your name\| 1,1, Uses your first name 2,2 Uses your proper name (Ms/Mr) or asks what you would like to be referred to as |
| 1. Explains study clearly | 0, 0, Gave no explanation\| 1,1, Explained 1 or two of the following: randomized controlled trial (control vs. no control, what will happen during trial, length or time \| 2,2, Explained randomized controlled trial (control vs. no control AND what will happen during trial AND length or time |
| 1. Recognize barriers to communication | 0, did not ask about hearing, 1 asked about or acknowledged participant’s hearing difficulty in indirect way, 2, asked about/acknowledged hearing difficulty in clear, nonjudgmental way |
| 1. Made adjustments to presentation to accommodate communication barrier | 0, did not adjust voice volume or positioning of self, 1, increased volume of speaking without collaborating with your or acknowledging the hearing challenge, 3, came up with a viable solution to help you hear better (e.g. increased volume, spoke more slowly, asked if family member was around, emailed study) AND acknowledged hearing challenged |
| Case 2: | |
| 1. Discusses risks appropriately | 0, 0, Did not discuss risks \| 1,1, Discussed risks BUT did not check for understanding or questions \| 2,2, Discussed risks AND checked for understanding or questions |
| 1. Discusses benefits appropriately | 0, 0, Did not discuss benefits\| 1,1, Discussed benefits BUT did not check for understanding or questions \| 2,2, Discussed benefits AND checks understanding or questions |
| 1. Ensures you understand the risks and benefits | 0, 0, Did not ask you to restate risks and benefits\| 1,1, Asks if you understand the risks and benefits but does not allow you to finish restating the risks and benefits \| 2,2, Has you restate the risks and benefits in your own words |
| 1. Invites questions | 0, 0, Tone/demeanor/language did not invite questions\| 1,1, Asked you had any questions BUT tone/demeanor/language did not encourage questions \| 2,2, tone/demeanor/language invited questions |
| 1. Acknowledges concerns | 0, 0, Did not acknowledge concern\| 1,1, Acknowledge concern BUT did not check for understanding or questions \| 2,2, Acknowledge concern AND checked for understanding or questions |
| 1. Reassure patient on confidentiality of information | 0, 0, Did not give much reassurance at all \| 1,1, Gave some reassurance but you still had questions OR gave an overwhelming volume of information \| 2,2, Gave all the reassurance you wanted/needed |
| 1. Maintain neutrality between family members | 0, 0, Did not maintain neutrality in tone, language, or body language \| 1,1, Maintained some neutrality, but favored communicating with one over the other \| 2,2, Maintained neutrality in tone, language, and body language |
| 1. Reaffirm that it is the patient’s decision whether or not to consent | 0, 0, Did not reaffirm that it was your decision \| 1,1, Reaffirmed that it was your decision BUT did not check for understanding \| 2,2, Reaffirmed that it was your decision AND checked for understanding |
|  |  |
| Case 3: | |
| 1. States that individual can withdraw from project at anytime | 0, 0, Does not state that individual can withdraw at any point \| 1,1, States that individual can withdraw but is vague or unclear    2 clearly States that individual can withdraw at any point |
| 1. States that individual’s decision will not affect care in anyway | 0, 0, Does not state that individual’s decision will not affect care in any way \| 1,1, States that individual’s decision will not affect care in any way but Is vague or unclear    Clearly States that individual’s decision will not affect care in any way |
| 1. Acknowledge the reality of the individual’s lived experience of racism | 0, 0, Does not acknowledge your statements about experience of racism\| 1,1, acknowledges your statement's about experience of racism BUT tone or behavior does not exhibit respect OR minimizes experience (e.g. “but that was back then” or ”that doesn’t usually happen”) \| 2,2, Acknowledge your statement's about experience of racism AND tone or behavior does exhibits respect |
| 1. States that individual does not need to decide immediately | 0, 0, Does not state that individual does not need to consent immediately \| 1,1, States that individual does not need to consent immediately but is vague or unclear    Clearly States that individual does not need to consent immediately |
| 1. States that “consent is voluntary” | 0, 0, Does not state that consent is voluntary \| 1,1, States that consent is voluntary BUT does not explain further , 2, states that consent is voluntary AND that it is participant’s decision AND participant may leave at any time |
